# What Is Frailty? Perspectives from Chinese Clinicians and Older Immigrants in New Zealand

**DOI:** 10.1007/s10823-021-09424-0

**Published:** 2021-04-08

**Authors:** Gary Cheung, Susan Gee, Hamish Jamieson, Ulrich Berger

**Affiliations:** 1 Department of Psychological Medicine, The University of Auckland, Private Bag 92019, Auckland Mail Centre, Auckland, 1142 New Zealand; 2 University of Otago and Canterbury District Health Board, Christchurch, New Zealand; 3 University of Otago, Christchurch, New Zealand

**Keywords:** Ethnicity, Frailty, Health professionals, Older people, Overseas Chinese

## Abstract

This qualitative study explores the meanings of frailty held by Chinese New Zealanders and Chinese health care professionals with the aim of identifying commonalities as well as potential differences. Two guided focus groups with Mandarin and Cantonese speaking older adults (*n* = 10), one individual interview with a English speaking older Chinese, and one focus group with Chinese New Zealand health care professionals (*n* = 7) were held to obtain views on frailty in older adults, followed by transcribing and a thematic qualitative analysis. Three main themes emerged: (1) Frailty is marked by ill-health, multiple chronic and unstable medical comorbidities, and is a linked with polypharmacy; (2) Frailty can involve physical weakness, decline in physical function such as reduced mobility or poor balance, and declining cognitive function; and (3) Frailty is associated with psychological and social health including depression, reduced motivation, social isolation, and loss of confidence. The perspectives of frailty that emerged are congruent with a multi-dimensional concept of frailty that has been described in both Chinese and non-Chinese medical research literature.

## Introduction

The flow of migrants from homelands in the Chinese mainland, Hong Kong, Macau and Taiwan has been described as a diaspora, with over 40 million people identifying as ethnically Chinese living in 148 other countries around the world (Poston and Wong 2016). This is based on a broad definition of overseas Chinese that includes sojourners maintaining their original citizenship (huaqiao, ), those naturalised to their host country (huaren, ), and descendants of Chinese (huayi, ) (Sluka et al. 2018). In the words of a Chinese poem: “wherever the ocean waves touch, there are overseas Chinese” (Poston and Wong 2016).

Australia, Canada, New Zealand, and the United States of America are primary destinations for Chinese immigration to western countries (Poston and Wong 2016). While there is an over century-long history of Chinese immigrating to New Zealand, there have been striking change in immigration patterns since 1986 following changes in immigration policies. By the 2013 census 4.3% of New Zealand’s population identified as Chinese compared to 0.8% in 1986, with almost three quarters being born overseas (Ho 2015; Statistics New Zealand 2014). New Zealand’s largest city Auckland is the primary destination for new immigrants, with two thirds of all people identifying as Chinese living in Auckland based on 2013 census data, and one in four Aucklanders identifying as Chinese (New Zealand Immigration 2018). This backdrop reinforces the importance of better understanding cultural differences in perspectives relating to health and illness and concomitantly in health-related behaviours (Heiniger et al. 2015).

New Zealand’s relatively young ethnic minority populations are ageing much faster than the general population, with an estimated expected increase of 260% in older Asian peoples expected from 2013 to 2036, compared to 60% for the population of older New Zealand Europeans (Statistics New Zealand, 2014). The issue of frailty amongst older Chinese New Zealanders will therefore be increasingly relevant. Frailty is an elevated state of risk or vulnerability characterised with loss of reserves including energy, physical ability, cognition and health (Fried et al. 2001). Older people with frailty are more vulnerable to a sudden decline in health and other negative outcomes in response to seemingly small trigger events or changes. Frailty increases with age as a consequence of age-related physiological declines, and it has been estimated that a quarter to a half of people aged 85 or over have frailty (Clegg et al. 2013). However, it is important to recognise that frailty varies in severity and it is not static, rather it can be made better or worse (Turner 2014). Identifying the frailty level of older people offers an opportunity to prevent or delay adverse outcomes by introducing appropriate care pathways, interventions, and individualised treatment plans (Bergman et al. 2007, Senior et al. 2014).

Previous international research studies of frailty in older Chinese people have predominately used quantitative methodologies (Chan et al. 2009; Chang et al. 2011; Hao et al. 2016; Lau et al. 2016; Ng et al. 2014; Woo et al. 2005; Woo et al. 2015; Wu et al. 2018). The perception of frailty within this group has not been previously explored. The aim of this study is to explore the understanding, meaning and experience of frailty with Chinese health care professionals (HCPs) and older Chinese New Zealanders. A focus group methodology was chosen for this qualitative research, which uses the group and its interaction as a way to gain information about frailty through the shared understanding amongst group members.

## Methods

### Settings and Participants

Three focus groups were conducted, the first with Chinese HCPs, the second with Cantonese speaking older Chinese, and the third with Mandarin speaking older Chinese. The Chinese HCPs were purposively recruited. They were clinicians working with older adults in the Auckland region. The Cantonese and Mandarin speaking older Chinese were recruited from one of the three Auckland day centres run by the Chinese Positive Ageing Charitable Trust (https://cpacharitabletrust.wordpress.com/centres/). The day centre runs weekly and provides group activities for 20 to 25 Cantonese and Mandarin speaking older Chinese.

Ethics approval was obtained from the University of Otago’s Human Ethics Committee (reference number 17/151.). Written consent was obtained from all study participants.

### Data Collection and Analysis

Data were collected regarding the participants’ age, gender, marital status, place of birth, number of years living in New Zealand, number of years of education and highest education. The focus groups were conducted between October and November 2018. Each focus group was facilitated by two to three bilingual (English-Chinese) researcher/research assistants with at least a master’s degree qualification. The facilitators used a topic guide (Appendix) to elicit ideas and discussion about frailty in later life. Focus group participants were asked to give examples of frailty, and further questions were used to explore why they thought an older person was frail. The focus groups were audio-recorded and professionally transcribed in English (HCP focus group) and Chinese (Cantonese and Mandarin speaking older Chinese focus groups). The transcripts were entered into NVivo, a computer assisted qualitative data analysis programme. The data were analysed by GC, a bilingual (English-Chinese) researcher, who co-facilitated two of the three focus groups. Themes were analysed using the thematic qualitative method described by Braun and Clarke (2006) whereby qualitative data is analysed using a six-phase process. The researcher familiarised himself with the data, catalogued recurring semantic concepts, searched for themes and reviewed the relevance of the themes compared to the full data set. GC is an academic old age psychiatrist but has not participated in any prior frailty research. He was not familiar with the frailty literature at the time of data collection and analysis, which minimised the bias of introducing existing frailty concepts into these processes.

## Results

There were a total of 18 participants in the three focus groups: HCP (*n* = 7; female 71.4%), Cantonese speaking older people (*n* = 6, female 100%), and Mandarin speaking older people (*n* = 4, female 75%). In addition, there was one English speaking older Chinese man recruited from the Chinese Positive Ageing Trust day centre. He was interviewed in English separately by one of the researchers. The demographic details of the older Chinese participants are shown in Table 1. The professional background of the HCP was nursing (*n* = 4), social work (*n* = 2) and mental health support worker (*n* = 1).

**Table 1:** Demographics of older Chinese participants in the two focus groups and individual interview

	**Older Chinese** ***N*** **= 11**
**Age** (median, range)	81 (68–85)
**Gender:** Female	9 (81.8%)
**Marital status**	
Married	5 (45.5%)
Divorced	1 (9.1%)
Widowed	5 (45.5%)
**Place of birth**	
China	8 (72.7%)
Hong Kong	1 (9.1%)
Macau	1 (9.1%)
The Philippines	1 (9.1%)
**First Language**	
Mandarin	4 (36.4%)
Cantonese	6 (54.5%)
English	1 (9.1%)
**No. of years in NZ** (median, range)	18.5 (5–40)
**Years of education** (median, range)	7.5 (0–15)
**Highest education**	
Primary school	4 (36.4%)
High school	3 (27.3%)
Tertiary education	2 (18.2%)
Nil	2 (18.2%)

The three focus groups lasted between 60 to 100 min. The individual interview was 26 min. Three main themes emerged from the thematic analysis on the understanding and description of frailty by Chinese HCPs and older people. In addition, the participants discussed the cultural expectation of ageing and frailty, and strategies to improve frailty in the community.

### Theme 1: Ill-Health, Medical Comorbidities and Polypharmacy

Both Chinese HCPs and older people reported ill-health, multiple/chronic/unstable medical problems and medical comorbidities are one of the most significant factors related to frailty. Pain (particularly leg pain) is another related factor. There is a commonly used Chinese term “ ”, which refers to “*sickness*” and “*pain*”. All of the Cantonese speaking older people listed metabolic syndrome “ ” (“*three-highs*”) as a condition that is associated with frailty. “*Three-highs*” is a commonly used Chinese medical term referring to hypertension, diabetes and hypercholesterolemia. Some of the Cantonese speaking older people thought frailty could lead to dizziness, poor immunity and further medical problems.

Some HCPs reported a relationship between polypharmacy and frailty. Many Cantonese speaking older people believed polypharmacy could lead to reduced immunity, ill-health and frailty. They thought medication side effects could be harmful for physical health because the “weakened” body is not able to process the medication. Most HCPs thought older Chinese prefer to take traditional Chinese medicine (TCM) over western medicine: “The Chinese (medicine) was more natural, I think, that’s what I think the older generation think of, you know. They are taking Chinese medication. There is no side effect or less side effect.” However, this opinion was not shared by the older Chinese people. They described a trusting relationship with their New Zealand general practitioners who prescribed them with western medication. They were also aware of the potential adverse interaction between TCM and western medication, and therefore they did not take both types of medication simultaneously.

### Theme 2: Physical Weakness, Decline in Physical and Cognitive Functioning

Our Chinese HCPs and older people frequently described the association between frailty with physical weakness and reduced energy. For example, the Mandarin speaking older people repeatedly used the following Chinese terms: “” (“*physically weak*”), “” (“*no strength*”), “” (“*not physically enough*”), and “” (“*powerless*”).

The HCPs and older Chinese also reported a reduction in the level of basic and complex activities of daily living. One Mandarin speaking older person mentioned: . , , , , , , .” (“*I feel old. Prior to 80 years old, I could do everything, I had confident and was not afraid of anything, could walk. Now I am older than 80 years old, I cannot do much and even have problem with walking*.” Another Mandarin speaking older person thought the reduction in activity level is due to low energy and strength: “, , .” (“*The main reason is lack of energy and strength, cannot do a lot of things or get going.*”*).* A number of HCPs and older Chinese were particularly concerned about reduced mobility. They described a range of symptoms and observations including poor balance, falls, slow gait, use of walking aids and wheelchair, and being bedbound. A few HCPS and Mandarin speaking older people described cognitive decline (such as forgetfulness, short term memory impairment, problems with thinking abilities, cognitive impairment and dementia) that could be associated with frailty.

### Theme 3: Association with Psychological and Social Health

Many of the HCPs and older Chinese believed depression could lead to poor physical health. For example, one Cantonese speaking older person said: “.” (“*I think mood has the greatest effect on health*.”). They also thought depression could lead to reduced interest in hobbies and activity level; and in extreme cases people could lose interest in themselves including their self-image and self-care.

The complex and inter-relationship between physical health, psychological health and social health was highlighted by the HCPs and older Chinese. For example, a HCP thought an older person who is emotionally struggling with ill-health and frailty could suffer from loneliness and social isolation; while a Cantonese speaking older person thought ill-health could be a result of loneliness and social isolation. One Cantonese speaking older person thought the lack of social support could lead to depression, reduced motivation and social isolation. In addition, a HCP thought older people could lose their confidence with frailty.

### Cultural Expectation of Ageing and Frailty

The HCPs discussed in length about how older Chinese often “expect” physical illness, functional impairment and frailty as part of the normal ageing process. For example, one HCP commented: “… sometimes they (older Chinese) are thinking that is the way of life, when you grow old become weak, and sickness is all coming.” Another HCP said: “… they see themselves as old and frail.” The HCPs commented that there is often a discrepancy between the clinician’s objective functional assessment and the older person’s own perception of their functionality. For example, one HCP mentioned: “… So even though they’re quite capable, to them they are old, they should feel old, they should look old, they should ask for help.”

This expectation of ageing and frailty was echoed by some Cantonese and Mandarin speaking older people. For example, there is a common Cantonese phrase “ ” “*(health) this year is not as good as the previous year*”. A Mandarin speaking older person believed that “, ” (“*Frailty is a process, faster for some people and slower for others*”).

The role of filial piety ( ) in Chinese culture was highlighted by several HCPs. In Confucian philosophy, filial piety is a virtue of respect for one’s parents, elders, and ancestors. One HCP commented: “… when you get older, you do expect your children to do more for you.” Another HCP also made a remark from her observation working as a social worker: “… so they not only expect family members to help them, they do expect professionals to help them as well.” However, one Mandarin speaking older person did not have such expectation on her children. Indeed, she did not want to be a burden on her children and would want to resolve any problems by herself. Another Mandarin speaking older person told the group that her son once asked her about her future and she replied:“… , , .” (“*If I became confused and no longer able to cook, I will go to a nursing home and will not give you any problem*.”). Similarly, a Cantonese speaking older person described her experience of depression and how she had to help herself and did not rely on other people: “, , , , , .” (“*sometimes when your mood is low, like thinking about family issues, you have to let things go because there is no other solution, it is very difficult to ask other people to help you, you need to help yourself.*”).

### Strategies to Improve Frailty in the Community

The HCPs overwhelmingly believed that targeted public health education is critical for understanding and reframing ageing and frailty for Chinese New Zealanders. They discussed ageing in Chinese often carries a rather negative image, for example the “loss of health, loss of friends, loss of family members”. They thought positive ageing education for older Chinese and their families could include the importance of valuing independence, leading a purposeful life, and adaptation to the ageing process. A number of the older Chinese expressed their appreciation of the care and support received from their family, friends and community groups. Some of them were also aware of the importance of physical activities, social interaction and having transport to access services in maintaining their physical and psychological well-being. A few people in the three focus groups thought finance is an important factor for health, for example, one HCP highlighted: “… actually because they got the money, they feel like they’ve got support if they want to go to see a doctor or something.” A couple of Mandarin speaking older people thought having their basic needs met (nutrition and warmth clothing) is important for their health: “, , .” (“*It will not work if there is not enough food, warmth clothing, or nutrition.*”).

## Discussion

Frailty can be viewed as a multi-dimensional concept covering physical, psychological, social and environmental factors (Markle-Reid and Browne 2003). To the best of our knowledge, this is the first qualitative study exploring the concept of frailty in Chinese. The three main themes emerged from our qualitative data are consistent with this multi-dimensional concept of frailty reported in both Chinese and non-Chinese cultures, including New Zealand’s Europeans and indigenous people. (Dury et al., 2018; Gee et al., 2019; Gee et al., in press; Puts et al., 2009, Teo et al., 2019). A previous systematic review, where majority of the studies were with Europeans, North Americans and Latin Americans, found the main factors associated with frailty are age, female gender, black race/color, schooling, income, cardiovascular diseases, number of comorbidities/diseases, functional incapacity, poor self-rated health, depressive symptoms, cognitive function, body mass index, smoking, and alcohol use (Mello et al. 2014). Many of these factors were also identified in our study and our results have therefore provided face validity for this approach to offer a meaningful way of talking about frailty for older Chinese New Zealanders. These results have enhanced our confidence in promoting frailty as a clinical syndrome that can be recognised and identified by Chinese clinicians and older immigrants.

The first theme “ill-health, medical comorbidities and polypharmacy” is supported by previous literature on frailty in Chinese**.** For example, a recent study found frail Chinese older people had higher prevalence of chronic conditions than the robust (Wu et al. 2018); other Chinese studies reported frailty was associated with medical comorbidities/chronic diseases (number ≥ 3) and polypharmacy (He et al., 2019; Woo et al. 2015). For frail older people, the burden of polypharmacy sometimes outweighs the burden of the original diseases (BPAC 2010b), and some medications may cause more harm than good (BPAC, 2010a). The result related to the metabolic syndrome was unexpected by our researchers who facilitated the Cantonese speaking older people group. However, it is consistent with the literature. Tang et al. (2013) found the level of frailty (as measured by the frailty index) increased with each cardiometabolic disorder in older Chinese. They concluded that cardiometabolic disorders are often present in the presence of other health deficits in frailty (Tang et al. 2013). An increase in inflammatory markers is associated with both frailty and metabolic syndrome; and these two conditions might share a common pathogenesis (Collerton et al. 2012; Kalyani et al., 2012; Li et al. 2019; Liu et al. 2013).

The Chinese-Canadian study of health and aging clinical frailty scale physician version (CSHA-CFS PV) provides a global impression of frailty by a physician following a comprehensive geriatric assessment. It has seven scores ranging from 1 (very fit) to 7 (severely frail) (Rockwood et al. 2005). The descriptor for “very fit” is *“robust, active, energetic, well-motivated and fit; these people commonly exercise regularly and are in the most fit group for their age”*; while “severely frail” is *“completely dependent on others for ADL, or terminally ill”*. Our second theme (physical weakness, decline in physical and cognitive functioning) is very much consistent with the CSHA-CFS PV descriptions and definitions of fitness and frailty. In addition, older Chinese with frailty are at risk for developing cognitive impairment and impairment in activities of daily living (He et al. 2019; Ma et al. 2020; Zhang et al., 2020a) and Malmstrom & Morley (2013) suggested cognitive frailty is going to be a widely accepted syndrome. In addition, a recent study found frail older Chinese with cognitive impairment had a higher rate of exhaustion, low activity, weakness and slowness than those without cognitive impairment (Li et al. 2019). The FRAIL scale has also been used in previous research involving older Chinese (Lau et al. 2016). It has five questions on fatigue *“Are you fatigued?”*, resistance *“Cannot walk up one flight of stairs?”*, aerobic *“Cannot walk one block?”*, illness *“Do you have more than 5 illnesses?”* and loss of weight *“Have you lost more than 5% of your weight in the last 6 months?”* (Morley et al. 2012).

Our third theme considered psychological and social health. Depression and mental health problems was found to be associated with frailty in older Chinese (Chang et al. 2011; Ma et al. 2020; Tian et al. 2018; Ye et al. 2018; Zhang et al., 2020b) and was an important contributor of frailty in the Singapore Longitudinal Ageing studies (Ng et al. 2014). It has been suggested people with depression may be more pessimistic about the progression of their health problems, perceive less control over their objective health and foresees a poorer prognosis of their current health complaints (Hong et al. 2004, Taylor and Brown, 1988). The association of social factors (e.g. unfavourable socioeconomic status, living alone, unmarried, loneliness, reduced social participation) and frailty was confirmed in previous Chinese studies (Sha et al. 2020; Wu et al., 2018; Ye et al., 2018; Ye et al. 2020). An indeed, Malmstrom and Morley (2013) developed a scale “SOCIAL” to screen for psychosocial risk factors for frailty: **S**adness, **O**utside activity, **C**ognition, Income adequacy, **A**ttachment to neighbour, **L**ethargy.

Woo et al. (2005) argued the concept of frailty should include broader environmental factors. Their study with older Canadian Chinese (age 70+) found frailty was associated with lack of social support network. They suggested this is likely that frail people are less likely to be active socially; but it is possible that active participation in society may delay the onset of frailty. The importance of social support network was also confirmed in a study of older Chinese people where being married was a protective factor of frailty (Woo et al. 2015).

It is interesting that education was identified as a strategy to improve the understanding of ageing and frailty by our Chinese HCPs. Frailty has been found to be associated with low education in previous studies with older Chinese (Chang et al. 2011; Woo et al. 2015; Wu et al. 2018); while no formal education was a significant contributor of frailty in the Singapore Longitudinal Ageing Studies (Ng et al. 2014).

A limitation of our study is that the collective term of Chinese in New Zealand subsumes diverse national, linguistic, and settlement backgrounds. There may be common differences amongst subgroups that could not be identified in small scale research. The frailty literature comparing different Chinese subgroups is very limited, but it has been shown that prevalence of frailty in north China and east China (13.4% and 13.6% respectively) is higher than that in Taiwan and Hong Kong (9.8%) (Tian et al. 2019). Another study concluded polypharmacy had a more significant contribution to frailty in Chinese living in Beijing compared to those living in Hong Kong; while being married and current alcohol use had a ‘protective’ effect in the Beijing cohort and Hong Kong cohort respectively (Woo et al. 2015).

Within the themes there was an interplay of Chinese and western values and perspectives around health and family roles in later life. Chinese HCPs and older people in our study differed in their opinions on a few issues. For example, HCPs thought older Chinese would prefer traditional Chinese medicine over western medicine, while older Chinese understood the important of potential adverse interaction between traditional Chinese and western medicine; HCPs reported a discrepancy between the clinician’s objective functional assessment and the older person’s own perception of their functionality; and lastly HCPs thought older Chinese expected their children to support them, a view which was not shared by some older Chinese participants. It is important to be aware that the experience of aging for older Chinese in New Zealand is a complex negotiation between inherited traditions and a western and globalized culture (Gee et al. 2002). Given the relatively small sample size, the different opinions by HCPs and older Chinese should not be generalised, but likely reflect differences in their unique personal experience of ageing, either directly or indirectly through observation. Another explanation is that HCPs in our study were asked to comment on frailty in Chinese people, but our older Chinese participants were recruited from a community centre and were relatively independent and not frail. Their differences, however, have reinforced the importance of person-centred care and support that takes into considerations and nurture individual identity (Brooker & Latham 2015). HCPs should be sensitive to cultural differences and not to make assumptions based on their own experience. To improve their communication and bridge expectation, the shared decision-making approach, where a patient is actively involved in decisions with their healthcare provider, can be applied to older people who present with medical comorbidities, chronic diseases and frailty (Cheung 2017).

## Conclusion

The main themes of frailty found in this qualitative study are largely consistent with that described in the Chinese and non-Chinese frailty literature, which is predominately from quantitative research. Our study provides support for some common underlying understandings of frailty existed across different ethnic groups, although the cultural interpretation of ageing and frailty should be included as part of the strategy to address frailty in different ethnic groups. Physical frailty is potentially reversible (Ng et al. 2014). We identified education could improve the recognition of frailty in Chinese. Public health, primary care planning and interdisciplinary approach are necessary to target the multi-dimensional nature of frailty in the community (Chang et al. 2011. Woo et al. 2015).

The findings of this study could be used to inform the development of Chinese specific health promotion materials on frailty to improve its recognition by older Chinese people and their families who would then seek medical advice prompting early identification, diagnosis and management of frailty by HCPs.

## Appendix: Summary of Focus Group Topic Guide

1. Initial introductions of people, purpose and confidentiality.
2. Engagement: What is frailty?

i. (Display two pictures) This is Ling. We haven’t seen Ling for a while and when we meet her we think gosh she is doing well. This is Ming we haven’t seen Ming for a while. When we see her we think gosh she is getting frail.
ii. What is it that make you think that someone is frail?
iii. What I want you to do is to write down three things that you think of when you think of an older person being frail. What’s involved in being frail? You’ll see you have a pad of sticky notes in front of you. We’d like you to use three sticky notes to write your three ideas, so each one on a separate page.
iv. Discussion of ideas

3. Exploration: What makes frailty better or worse?
  i. So we have some older people who are frail, they xxx and may xxx [summarise consensus themes and non-consensus themes]. What might make frailty worse for them?
  ii. What might make things better for them?
4. I just want to focus in on one of the topics that was brought up /that didn’t get brought up: things like medicine that people might take – they might get them prescribed, or they might get them from the chemist shop or the health shop, or make them up themselves.

i. When you think about all those different types of medicines, what do you think about medicines as a help for people who are frail?
ii. [If it isn’t raised] Are there times when you think medicines could make things worse for someone who is frail?
iii. So what would you think if someone looked through all the different medicines you were on and gave you advice about the ones that might do more harm than good for someone who was frail?

5. To finish, summarize with confirmation

i. Does that seem an ok summary?
ii. All things considered question “Of all the things we discussed, what to you is the most important, what do you think we should really take way?”
iii. Have we missed anything?
