# Correction to: What Is Frailty? Perspectives from Chinese Clinicians and Older Immigrants in New Zealand

**DOI:** 10.1007/s10823-021-09429-9

**Published:** 2021-04-23

**Authors:** Gary Cheung, Susan Gee, Hamish A. Jamieson, Hans Ulrich Bergler

**Affiliations:** 1 Department of Psychological Medicine, The University of Auckland, Private Bag 92019, Auckland Mail Centre, Auckland, 1142 New Zealand; 2 University of Otago and Canterbury District Health Board, Christchurch, New Zealand; 3 University of Otago, Christchurch, New Zealand

**Correction to: Journal of Cross-Cultural Gerontology**

10.1007/s10823-021-09424-0

Correction is needed to the names of the 3rd author and 4th author in the original publication. The correct author names are presented in this Erratum.

